# Revisiting the role of ascorbate oxidase in plant systems

**DOI:** 10.1093/jxb/erae058

**Published:** 2024-02-16

**Authors:** Ifigeneia Mellidou, Angelos K Kanellis

**Affiliations:** Institute of Plant Breeding and Genetic Resources, Hellenic Agricultural Organization ELGO-DIMITRA, 57001 Thessaloniki, Greece; Group of Biotechnology of Pharmaceutical Plants, Laboratory of Pharmacognosy, Department of Pharmaceutical Sciences, Aristotle University of Thessaloniki, 541 24 Thessaloniki, Greece; University of Birmingham, UK

**Keywords:** Antioxidants, hormone homeostasis, oxidative stress, redox state, ripening

## Abstract

Ascorbic acid (AsA) plays an indispensable role in plants, serving as both an antioxidant and a master regulator of the cellular redox balance. Ascorbate oxidase (AO) is a blue copper oxidase that is responsible for the oxidation of AsA with the concomitant production of water. For many decades, AO was erroneously postulated as an enzyme without any obvious advantage, as it decreases the AsA pool size and thus is expected to weaken plant stress resistance. It was only a decade ago that this perspective shifted towards the fundamental role of AO in orchestrating both AsA and oxygen levels by influencing the overall redox balance in the extracellular matrix. Consistent with its localization in the apoplast, AO is involved in cell expansion, division, resource allocation, and overall plant yield. An increasing number of transgenic studies has demonstrated that AO can also facilitate communication between the surrounding environment and the cell, as its gene expression is highly responsive to factors such as hormonal signaling, oxidative stress, and mechanical injury. This review aims to describe the multiple functions of AO in plant growth, development, and stress resilience, and explore any additional roles the enzyme might have in fruits during the course of ripening.

## Teaser

This review aims to investigate the multifaceted regulatory mechanisms of ascorbate oxidase in different plant species/tissues beyond the common perspective of the trade-off between ‘beneficial’ antioxidants and ‘harmful’ reactive oxygen species.

## Introduction

Ascorbic acid (AsA) is highly prevalent in all plant tissues, where it acts as the master contributor to the cellular redox state (Smirnoff, 2000). While most AsA is in the cytoplasm, a smaller but significant proportion is found in the apoplast (Noctor and Foyer, 1998). This apoplastic AsA is deemed to serve as the initial defense against external stimuli and likely plays a pivotal role in mediating responses to stresses that generate an oxidative burst (Pignocchi and Foyer, 2003; Sanmartin *et al*., 2003; Fotopoulos and Kanellis, 2013). Although it can generate radicals and act as a pro-oxidant when in high concentrations, it primarily functions as an antioxidant, eliminating reactive oxygen species (ROS) and supporting cellular redox balance in various cell compartments, including chloroplasts, peroxisomes, and mitochondria, where ROS highly accumulate during fruit ripening and postharvest (Wheeler *et al*., 2015; Arabia *et al*., 2024). Additionally, AsA is able to regenerate the antioxidant functions of other compounds, such as α-tocopherol (Foyer and Shigeoka, 2011), to facilitate iron uptake by reducing Fe^3+^, and to act as a coenzyme for key enzymes involved in processes such as photoprotection through violaxanthin de-epoxidase and the synthesis of plant hormones (Arabia *et al*., 2024). It has also been linked to the control of cell division and expansion, cell differentiation, and the regulation of programmed cell death (Mellidou *et al*., 2017). Overall, AsA is engaged in numerous physiological processes, especially those involving critical redox changes, covering plant development, fruit ripening, and senescence.

In the apoplast, ascorbate oxidase (AO; EC 1.10.3.3) serves as a critical enzyme involved in the oxidation of AsA to the less stable radical monodehydroascorbate (MDHA), which rapidly disproportionates to generate dehydroascorbate (DHA) and AsA (Smirnoff, 2000). In this reaction, AsA, which is transported to the apoplast by plasma membrane-associated transporters, is used as the electron donor for AO to reduce O_2_ to H_2_O (De Tullio *et al*., 2013). This pathway can function to dissipate the excess reducing power from the chloroplasts through the cytochrome b561 (cyt b) chain, which is able to transfer electrons directly across the plasma membrane from cytoplasmic AsA to apoplastic MDHA (Foyer *et al*., 2020).

AO is mostly found in rapidly growing plant tissues such as ovaries and green immature and ripe fruits (Liso *et al*., 1988; Lin and Varner, 1991; Esaka *et al.*, 1992; Moser and Kanellis, 1994; Diallinas *et al*., 1997; Al-Madhoun *et al.*, 2003; Liso, 2004; Sanmartin *et al*., 2007; Chatzopoulou *et al*., 2020). It is highly abundant in the family of cucurbits, in which its expression peaks just before the climacteric rise during fruit development (Sanmartin *et al.,* 2007). AO, associated with cell wall and hormone-mediated [auxin, ethylene, and jasmonate (JA)] processes, has a dynamic role in signal perception and transduction (De Tullio *et al*., 2007, 2013), with its expression being under tight transcriptional and translational control (Esaka *et al*. 1992; Diallinas *et al*., 1997; Sanmartin *et al*., 2007).

Since alterations in *AO* gene expression and AO enzyme activity can influence the redox state of AsA, it can potentially affect plant tolerance to oxidative stress (Pignocchi *et al*., 2003; Sanmartin *et al*., 2003; Fotopoulos *et al*., 2008). As AO can consume the AsA pool, it has been assumed to weaken plant defenses, and it is generally considered as an enzyme that carries no apparent advantage. Nonetheless, this oversimplified assumption overlooks the fact that that *AO* transcript levels are finely regulated in response to developmental and environmental stimuli and can vary in a species-dependent manner (Pignocchi and Foyer, 2003; Sanmartin *et al*., 2007). Our recent findings showed that melon fruits with high AsA contents due to *AO* silencing undergo accelerated ripening through enhanced ethylene production, as well as smaller fruit size with more cells of smaller size (Chatzopoulou *et al*., 2020). Another positive role of AO has been demonstrated by Balestrini *et al*. (2012), who revealed the unexpected involvement of AO during the symbiotic interaction of *Lotus japonicus* with beneficial microorganisms. Although the concept of apoplastic AO at the heart of the redox signaling hub has been well established, knowledge of its transcriptional and post-transcriptional regulation in response to various stimuli or across different species and developmental stages remains rudimentary. This review aims to discuss the intriguing and diverse functions of AO in plants, paving the way to new hypotheses and research avenues towards engineering future crops enriched in AsA.

## Apoplastic ascorbate oxidase is widespread within the plant kingdom

Although AO was initially discovered in cabbage (Szent-Györgyi, 1931), it mostly became a popular subject of research after its biochemical characterization in plant species of the Cucurbitaceae family (De Tullio *et al*., 2013). With recent advances in genomics, several *AO* gene orthologs have been identified across plant species such as *Arabidopsis thaliana*, *Cucurbita* spp., *Glycine max*, *Oryza sativa*, *Zea mays*, *Sorghum bicolor*, *Gossypium hirsutum*, and *Solanum lycopersicum* (De Tullio *et al*., 2013; Xin *et al*., 2016; Li *et al*., 2017). Overall, the *AO* gene family is relatively small, with a few gene copies showing detectable expression, which mostly differs under stress or through the course of development and ripening (Ohkawa *et al*., 1989; Diallinas *et al*., 1997; Sanmartin *et al*., 2007; Batth *et al*., 2017; R. Stevens *et al*., 2018). Putative *AO* gene sequences have also been detected in lower plants such as the lycophyte *Selaginella moellendorffii*, the moss *Physcomitrella patens*, and green algae of the genera *Chorella* and *Chlamydomonas* (De Tullio *et al*., 2013), suggesting its early emergence in the plant lineage and probably its vital roles for plant survival and adaptation. Nonetheless, the fact that the non-photosynthetic plant *Cuscuta reflexa* (which is able to synthesize AsA from the l-galactose pathway) has no AO activity indicates either that this enzyme is not crucial for plant survival or that its functions can be, at least partly, accomplished in alternative ways (Tommasi *et al*., 1990).

AO is a member of the blue copper oxidase family (Messerschmidt and Huber, 1990), responsible for the oxidation of AsA to DHA that takes place in the apoplast, with the simultaneous safe reduction of molecular O_2_ to water without the generation of ROS (Arrigoni *et al*., 1992; Smirnoff, 2000). In turn, DHA is transported to the cytosol with high affinity across the membrane and is replaced by AsA to maintain a constant redox flux (Horemans *et al*., 2000). Several studies in transgenic plants have demonstrated the apoplastic location of AO, associated with altered AsA redox states, and shifts in their ability to withstand oxidative stimuli (Pignocchi *et al*., 2003; Sanmartin *et al*., 2003; Fotopoulos *et al*., 2006). In this context, apoplastic AO has been implicated in plant growth and development, or in stress perception and downstream signaling events (Foyer and Noctor, 2005, 2011; Sanmartin *et al*., 2007; De Tullio *et al*., 2013). High AO activity can be detected in young fruits and roots, especially in the quiescent center, shoot apices, petals, and ovules (Lin and Varner, 1991; Esaka *et al*., 1992; Kerk and Feldman, 1995; Diallinas *et al*., 1997; Liso *et al*., 2004; Sanmartin *et al*., 2007; Matamoros *et al.*, 2010), but it is nearly absent from seeds (Arrigoni *et al*., 1992; Terzaghi and De Tullio, 2023).

Apart from its tissue specificity, AO activity also varies remarkably among different plant species. Hence, plant species of the Cucurbitaceae family (i.e. cucumber, melon, pumpkin, and squash) exhibit high AO activity (Liso *et al*., 1988; Lin and Varner, 1991; Esaka *et al*., 1992; Moser and Kanellis, 1994; Bin Saari *et al*., 1995; Diallinas *et al*., 1997; Al-Madhoun *et al*., 2003; Liso, 2004; Chatzopoulou *et al*., 2020), whereas Solanaceae species, such as tobacco, tomato, and bell pepper, show lower activities (Yahia *et al.*, 2001; Sanmartin *et al*., 2003; Garchery *et al*, 2013). The higher AO activity reported in Cucurbitaceae species seems to be associated with rapid fruit growth in these species, whereas there is no clear evidence for the participation of AO in fruit growth in tomato. However, AO in tomato seems to serve as a key regulator for several metabolic processes, providing the trigger to slow or adjust the metabolism under stressful environments (R. Stevens *et al*., 2018). In species such as *Z. mays*, *G. hirsutum*, *Beta vulgaris*, and *Hordeum vulgare* (Kerk and Friedman, 1995; Tamás *et al.*, 2006; Li *et al*., 2017; Skorupa *et al*., 2022), AO activity is also relatively low. In the roots of *Z. mays*, AO regulates AsA content to maintain the cells of the quiescent center in a state of reduced mitotic activity (Kerk and Friedman, 1995). Therefore, it is conceivable that the steady state of AO activity can either support or arrest growth in a species- and tissue-specific manner. Although it is not clear why some species have higher AO activities to support their needs, it is notable that the changes in the apoplastic AsA levels mediated by AO do not necessarily lead to changes in the size of the whole-tissue AsA pool (Pignocchi *et al*., 2003; Foyer *et al*., 2020), further supporting the notion that AO operates alongside and together with other oxidases to orchestrate tailored defense responses.

## Regulatory mechanisms and crosstalk in ascorbic acid oxidation: transcriptional, post-transcriptional, and epigenetic control

It has been well documented that the transcript levels of *AO* are controlled by several transcriptional and post-transcriptional factors (Batth *et al*., 2017), with *AO* expression generally being stimulated by growth promoters such as indole-3-acetic acid (IAA) (Pignocchi *et al*., 2003) or JA (Sanmartin *et al*., 2007), and repressed by growth suppressors such as salicylate (SA) (Pignocchi *et al*., 2003; Sanmartin *et al*., 2007) or wounding (Diallinas *et al*., 1997; Sanmartin *et al.*, 2007). Recent findings in Arabidopsis highlighted a transcription factor, DEFECTIVE IN TAPETAL DEVELOPMENT AND FUNCTION 1 (TDF1), as a negative regulator of AsA accumulation via regulating AsA oxidation through the activation of expression of *Skewed5* (*SKU5*) *SIMILAR 18* (*SKS18*); the SKS18 enzyme has AsA oxidation activity similar to that of AO (Wu *et al.*, 2023). In turn, TDF1-regulated AsA is able to control cell division and cell differentiation in the tapetum by controlling redox homeostasis. Besides transcription factors, small non-coding RNAs (miRNAs) are able to regulate gene expression directly at the post-transcriptional level. Hence, in bread wheat, the putative regulation of various *AO* isoforms by nine different miRNAs was revealed by targeting drought- and heat-stress-related transcription factors (Madhu *et al.*, 2023). Similarly, miR4415, a legume-specific miRNA, was associated with cold acclimation of *Ammopiptanthus nanus* by targeting AO and thus controlling the redox state of the apoplast (Zhu *et al*., 2022), further supporting the relevance of miRNAs in stress responsiveness.

On the other hand, early studies in pumpkin (Esaka *et al*., 1992) and maize roots (Kerk and Feldman, 1995) illustrated that the expression levels of *AO* did not necessarily correlate with AO enzyme activity, pointing to a translational or post-translational regulation mechanism. Previously, a similar discrepancy between enzyme activities and transcript levels of AsA recycling genes was reported in tomato fruits during ripening (Mellidou *et al*., 2012). For both AsA oxidation and AsA recycling genes, it is considered that there is a strong but temporary control over their expression, as the AsA redox balance is tightly regulated to support ROS scavenging.

Recently, a systems biology approach was used in tomato fruit in a study reporting that the activity and expression of genes regulating the AsA redox state correlated with a limited number of metabolites, probably reflecting post-transcriptional regulation (R.G. Stevens *et al*., 2018). Interestingly, metabolites able to differentiate *AO-* and *monodehydroascorbate reductase* (*MDHAR*)*-*silenced lines included the amino acids alanine and tyrosine (lower in *AO-*silenced lines) and the hexose sugars glucose and fructose (higher in *AO-*silenced lines). Additionally, there was a remarkable transcriptome inversion between *AO*-silenced lines on the one hand and *MDHAR/GLDH*-silenced lines on the other hand. A candidate signal molecule triggering this redox regulation is MDHA, which has a short life and is probably involved in protein modification due to proton transfer when AsA is recycled, or in the hyperpolarization of the plasma membrane that triggers cell enlargement in Cucurbitaceae. In line with this, AsA shows a negative post-transcriptional feedback control over its biosynthesis through *GDP-**l**-galactose phosphorylase* (*GGP*), influencing ribosome density on the 5ʹ untranslated region of the gene (Laing *et al*., 2015).

Epigenetic marks, including DNA methylation in gene promoters and exons, as well as histone modifications, contribute significantly to gene regulation in response to environmental stimuli. Although the AsA epigenetic regulation has been poorly investigated, recently, DNA methylation patterns along with *AO* expression levels under salt and drought stress were identified in *B. vulgaris* and its halophytic ancestor *Beta maritima* (Skorupa *et al*., 2022). Results highlighted remarkable differences in CpG islands of the promoter region of different *AO* orthologs that are responsible for silencing *AO* expression, which were dependent on both the type of stress and the plant species. In our previous study examining potato tubers grown in diverse environments, a notable demethylation took place in the promoter and intron regions of *GGP* (Boutsika *et al*., 2023). These modifications in the methylome appeared to be linked to differences in the AsA pool size of potato tubers. In tomato, a histone lysine demethylase, known for its role in removing methylation specifically from histone 3 lysine 27 (H3K27), is believed to trigger fruit ripening by controlling the expression of various transcription factors and genes associated with ethylene metabolism. In conclusion, understanding the epigenetic processes governing AsA biosynthesis, recycling, and oxidation could be pivotal in developing strategies to biofortify crops.

## Ascorbate oxidase is a principal redox regulator

**Fig. 1.**
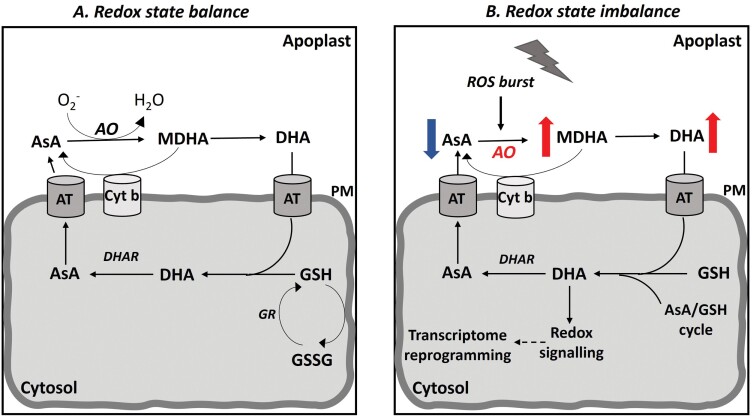
A proposed model for the role of ascorbate oxidase (AO) in regulating the apoplast redox state in response to oxidative stimuli. (A) In the absence of stress, AO functions in the apoplast to maintain balanced levels of reactive oxygen species (ROS) by regulating the redox state balance. Ascorbic acid (AsA) is transported from the apoplast to the cytosol and vice versa by plasma membrane-associated carriers (AT). (B) Upon oxidative stimuli, the induction of ROS leads to the enhanced transcription of *AO* genes, which results in the depletion of the AsA pool and the production of its oxidative forms in the apoplast. These events can cause a redox state imbalance in the apoplast. In turn, the redox equilibrium controlled by AO serves as a strong signal activating antioxidant metabolism. The activation of defense pathways by transcriptome reprogramming would determine whether the redox balance can be restored. Red arrows indicate up-regulation, the blue arrow indicates down-regulation, and the dashed line indicates signalling. AT, plasma membrane-associated carriers; Cyt b, cytochrome b561; DHA, dehydroascorbate; DHAR, dehydroascorbate reductase; GR, glutathione reductase; GSH, glutathione; GSSG, glutathione disulfide; MDHA, monodehydroascorbate; PM, plasma membrane.

Over the past decades, our understanding of the interplay between antioxidants and oxidants has largely progressed. The fine-tuning of the redox state can serve as an effective way to stimulate local and systemic responses during development and ripening, or in response to various exogenous stimuli (De Tullio *et al*., 2013). AO, being present in the majority of plant species (both photosynthetic and non-photosynthetic) and in the cell walls of all developmentally active tissues, has a key function in the regulation of the apoplastic redox state to maintain balanced ROS levels (Pignocchi *et al*., 2003; Sanmartin *et al*., 2003, 2007; Fotopoulos *et al*., 2008) (Fig. 1). The regulation of the AsA redox state and, conceivably, ROS accumulation is associated with the fact that AO uses O_2_ to catalyze the oxidation of AsA to the unstable molecule MDHA, which in turn disproportionates to yield DHA and AsA (Smirnoff, 2000). Transgenic tobacco (*Nicotiana tabacum*) plants overexpressing the cucumber apoplastic *AO* showed reduced water loss and stomatal conductance, as well as elevated abscisic acid (ABA) and H_2_O_2_ accumulation, suggesting a possible DHA-mediated mechanism of signal transduction associated with stomatal dynamics (Fotopoulos *et al*., 2008). On the other hand, the fate of AsA then AO activity is elevated is arguable. For instance, although the overexpression of *AO* in tobacco significantly reduced the AsA and glutathione (GSH) redox states, the total AsA pool size was not depleted (Pignocchi *et al*., 2003; Sanmartin *et al*., 2003), suggesting that the redox state is dynamically tuned. This function can be very important, especially under stressful environments, where sudden alterations in the redox status can help plants sense stimuli and modulate their defense responses, as the first events take place at the plasmalemma interface (Fotopoulos and Kanellis, 2013). Indeed, it has been previously found that transcript levels of *AO* were stimulated by light in tobacco and *Cucurbita pepo* (Pignocchi *et al*., 2003; De Tullio *et al.*, 2007), by wounding and heat in melon (Sanmartin *et al*., 2007), or upon inoculation with plant-growth-promoting rhizobacteria (PGPR) in tomato (Mellidou *et al*., 2021; Papadopoulou *et al*., 2023).

Apart from the obvious trigger of AO activity upon abiotic or biotic cues, it is reasonable to assume that alterations in the expression of apoplastic *AO*, leading to modifications in the redox state, can modify the degree of tolerance or vulnerability to oxidative stress, as highlighted in studies with transgenic plants (Table 1), most which used *N. tabacum*. The general consensus is that *AO* overexpression leading to enhanced stress vulnerability is associated with the down-regulation of the general antioxidant metabolism (mostly AsA recycling), whereas *AO* suppression leading to enhanced stress tolerance is related to its protective function over the apoplastic redox balance, preventing excessive H_2_O_2_, which is typically produced under stress conditions. *AO* overexpression in tobacco plants leading to a lower apoplastic AsA redox state enhanced plant sensitivity to ozone (Sanmartin *et al*., 2003) as well as to multiple oxidative stimuli (methylene blue as a singlet O_2_ inducer, or methyl viologen as a superoxide inducer) by suppressing the transcript levels of the AsA recycling genes (Fotopoulos *et al*., 2006). The transgenic plants exhibited a substantial increase in leaf injury, accompanied by a decline in CO_2_ assimilation, without alterations in leaf morphology (Sanmartin *et al*., 2003). Nonetheless, in a similar study, an ozone-sensitive clone of *Trifolium repens* had higher AsA redox status compared with a tolerant clone, suggesting that high AsA levels cannot always be sufficient to trigger ozone tolerance (D’Haese *et al*., 2005). A possible explanation should be sought in the role of AsA as a signaling molecule, rather than as a bulk ROS detoxifier (Bellini and De Tullio, 2019). In this context, it can be assumed that external applications of AsA to plants can stimulate their short-term tolerance, whereas in the long run, elevated AsA levels can make AsA signaling inefficient to support redox balance. Surprisingly, there were no remarkable differences in the transcript levels of AsA recycling genes between *AO*-overexpressing lines and wild-type plants under optimum conditions, highlighting the absence of a persistent shift in the antioxidant metabolism (Fotopoulos *et al*., 2006). In line with these findings, no remarkable differences in the phenotypes of *AO* sense and antisense Arabidopsis plants grown under normal conditions were evident compared with wild-type plants (Yamamoto *et al*., 2005). Conversely, upon salt stress, *AO* antisense plants exhibited increased salt tolerance, attributed to the reduced accumulation of H_2_O_2_ and the maintenance of a high redox state for both symplastic and apoplastic AsA. As a general rule, it has been demonstrated that *AO*-overexpressing tobacco lines show a reduced capacity to trigger the antioxidant defense mechanism to combat oxidative damage (Fotopoulos *et al*., 2006). Consistently, overexpression of *AO* leading to a drop in the apoplastic AsA redox state can lead to delayed dark-induced senescence, correlating with enhanced antioxidant enzyme activities and improved AsA recycling (Fotopoulos and Kanellis, 2013). The activity of AO seems to have a pivotal role in the activation of protein sensors participating in the acclimation of leaves in response to light. Hence, apart from the reduction in AsA redox state, under high light conditions, the suppression of *AO* in tobacco leads to higher photosynthetic rates and metabolomic reprogramming related to fatty acids, amino acid metabolism, and protein turnover (Karpinska *et al*., 2018), suggesting that AsA has vital functions related to the acclimation of plants to high light.

**Table 1.** Summary of transgenic efforts to modify *AO* expression in different plant species, and its effect on plant phenotype and physiology under normal and stressful conditions

Species	Gene modification	Conditions	Major effects compared with wild-type plants	Reference
**Normal**				
*Nicotiana tabacum*	Overexpression (*Cucurbita maxima*)	No stress	High growth rate, increased oxidation of AsA pool	Pignocchi *et al*. (2003)
	Overexpression (*Cucumis sativus*)		High H_2_O_2_ and ABA levels, low ROS scavenging enzyme activity, partial stomatal closure	Fotopoulos *et al*. (2008)
	Overexpression		No effect	Yamamoto *et al*. (2005)
	Antisense		Delay in flowering	Yamamoto *et al*. (2005)
*Cucumis melo*	Silencing		High fruit AsA content, increased ethylene production rate, and a dramatic arrest in fruit growth	Chatzopoulou *et al*. (2020)
**Abiotic stress**				
*Nicotiana tabacum*	Overexpression (*Cucumis sativus*)	Ozone	Reduced AsA redox state, increased GSH redox state, greater foliar injury	Sanmartin *et al.* (2003)
	Overexpression	Salinity	Very low redox state of symplastic and apoplastic AsA	Yamamoto *et al*. (2005)
	Antisense	Salinity	Low levels of H_2_O_2_ accumulation; high rates of germination, photosynthetic activity, and seed yields	Yamamoto *et al*. (2005)
	Overexpression	Oxidative factors	Reduced ability to stimulate defense mechanisms, i.e. AsA recycling	Fotopoulos *et al*. (2006)
	Overexpression	Dark-induced senescence	Reduced chlorophyll loss, enhanced antioxidant enzyme activities, and increased expression of AsA recycling genes	Fotopoulos and Kanellis (2013)
	Overexpression (*Cucurbita maxima*)	Excess light	Significant changes in amino acid levels	Karpinska *et al*. (2018)
	Antisense	Excess light	Higher photosynthetic rates	Karpinska *et al*. (2018)
*Solanum lycopersicum*	RNA silencing	Water deficit	Higher photosynthetic rates	Zhang *et al*. (2011)
*Solanum lycopersicum* (cherry)	RNA silencing	Water deficit	Improved yield, high stomatal conductance, high sugar content, increased fruit growth rate	Garchery *et al*. (2013)
	RNA silencing	Salt stress	Higher plant height, stem diameter, flower numbers, and fruit yield, as well as improved fruit lycopene and sugar contents	Abdelgawad *et al*. (2019)
Arabidopsis	Overexpression (*Ammopiptanthus nanus*)	Cold stress	Increased root and hypocotyl length	Zhu *et al*. (2023)
**Biotic stress**				
*Nicotiana tabacum*	Overexpression	*Botrytis cinerea*	Enhanced susceptibility to fungal infection	Fotopoulos *et al*. (2006)
	Overexpression (*Cucurbita maxima*)	*Pseudomonas syringae*	Loss of auxin sensitivity, low AsA recycling activities, and higher susceptibility to the pathogen	Pignocchi *et al*. (2006)
	Transient silencing	Cucumber mosaic virus	Reduced virus accumulation in systemic leaves	Kumari *et al*. (2016)
	Virus-induced gene silencing	Cucumber mosaic virus	Expansion of the spreading area of the virus	Ahmed *et al*. (2024)
Arabidopsis	Overexpression (*Cucumis sativus*)	Cucumber mosaic virus	No effect	Kumari *et al*. (2016)

Apart from tobacco, some notable transgenic efforts to unravel the way in which AO exerts its functions have been performed in tomato plants. For instance, under drought stress, *AO*-suppressed transgenic tomato plants had higher photosynthetic rates and stomatal conductance (Zhang *et al*., 2011), similar to findings in tobacco (Table 1). Additionally, *AO* RNA interference (RNAi) lines with reduced AO activity exhibited improved yield and enhanced transport of assimilates from source-to-sink tissues under water-deficit stress (Garchery *et al*., 2013). This correlation between AO activity and resource allocation of assimilates has also been demonstrated in Arabidopsis, in which *AO* antisense suppression led to higher seed yield upon stress (Yamamoto *et al*., 2005). This further supports the notion that when resources are limiting, apoplastic sucrose levels are reduced, correlating with the decreased AO activity in fruit. The lower fruit sucrose levels can in turn facilitate sucrose uptake from the phloem, leading to faster conversion of sucrose to hexose during fruit ripening (Garchery *et al*., 2013). Notably, no remarkable differences in fruit total AsA levels were found between transgenic and wild-type plants, pointing to the rapid feedback regulation of the AsA pool size, at least in tomato fruit (Mellidou *et al*., 2012). Under moderate and high salt stress, tomato transgenic lines with lower *AO* transcript levels were also more tolerant, exhibiting improved fruit yield, fruit quality, and plant performance compared with both wild-type and *AO*-overexpressing lines (Abdelgawad *et al*., 2019). This increase in fruit yield could be attributed to the higher hexose-to-sucrose ratio in the apoplast of the transgenic lines.

A recent study in *A. nanus* underlined the effect of the duration of stress on *AO* transcript levels, revealing that *AO* expression levels and enzyme activity decreased under short-term (24 h) cold stress, but they increased under long-term (7 d) stress (Zhu *et al.*, 2023). These findings suggest that the expression of *AO* genes is dynamically regulated by O_2_ levels and can change rapidly following the need for ROS detoxification. In particular, it was revealed that the expression levels of *AO* genes and enzyme activity reduced temporarily under short-term osmotic and low-temperature stress (for 1 d), but the expression of some *AO* gene orthologs was enhanced under 7 d of cold stress. Furthermore, functional validation of the *AnAO5* gene revealed that its overexpression in Arabidopsis enhanced the tolerance of plants to low temperature, suggesting that *AnAO5* is probably involved in cold acclimation in *A. nanus* by regulating the AsA redox state of the apoplast (Zhu *et al*., 2022).

In view of the above, it can be concluded that our understanding of the role of AO in stress tolerance remains patchy at best, as it can greatly vary across different plant species, tissues, and developmental stages, as well as with the magnitude and duration of stress. Given that plant cells show diverse responses to environmental stimuli and through the course of development, the use of the rapidly progressing spatial or single-cell transcriptomic technologies will probably accelerate the ongoing research in the field of plant stress tolerance, offering exciting new opportunities for biotechnological applications (Seyfferth *et al*., 2021).

## Ascorbate oxidase has a key role in plant–pathogen interactions

To counteract plant pathogens (viruses, fungal, bacteria, and herbivorous insects), plants have developed complicated yet sophisticated strategies, which include the induction of an oxidative burst resulting in ROS accumulation, and the stimulation of the antioxidant defense mechanism; both mechanisms acting in concert as a ‘double-edged sword’ to ameliorate stress (Boubakri *et al*., 2016). AsA has long been considered to serve as a key component in plant defense mechanisms against many pathogens (Mittler *et al*., 2004). Since AO is localized in the apoplast, it has a pivotal role in priming plants to respond to stress stimuli through finely controlling the apoplastic AsA redox state and H_2_O_2_ levels (Foyer and Noctor, 2005; Fotopoulos *et al.,* 2008), affecting cellular signaling cascades and hormonal responses. Priming against biotic factors can occur through the presence of various chemical compounds that are able to activate defense responses to ameliorate injury to the plant’s tissues. In this context, the application of AO to leaves was found to stimulate the systemic defense response of rice roots against the root-knot nematode *Meloidogyne graminicola* (Singh *et al.*, 2020b), and of sugar beet roots against the cyst nematode *Heterodera schachtii* (Singh *et al*., 2020a). Although the infection strategies of the two nematodes are different, the protective function of AO is similar, involving the activation of antioxidant activity, and, notably, did not involve a trade-off with plant growth and development due to the oxidation of the AsA redox state. The AO-induced defense mechanism seems to be related to hormonal reprogramming, with a temporary accumulation of JA, ethylene, and SA in the roots. Additionally, the early up-regulation of *AO* after infection of *Nicotiana benthamiana* with cucumber mosaic virus (CMV) was associated with viral movement proteins, leading to disturbed formation of functional AO dimers that are necessary for the spread of virus to adjacent cells, thus diminishing defense responses (Kumari *et al*., 2016) (Table 1). By contrast, silencing of another *AO* gene in tobacco was found to increase the severity of CMV infection, indicating that other members of the *AO* family may function as resistance factors against this disease (Ahmed *et al*., 2024). Interestingly, *AO* was also found to be among the most strongly differentially regulated genes in response to *Verticillium* wilt between tolerant and susceptible genotypes, clearly pointing to its beneficial contribution to the defense response against plant pathogens (Guo *et al*., 2017). On the other hand, although a short-term induction of *AO* expression can be advantageous for the plant immune system initially, the long-term shift towards a more oxidized AsA redox state may be harmful. This is consistent with the increasing susceptibility of transgenic tobacco plants overexpressing *AO*, which are unable to efficiently control disease progression compared with wild-type plants (Fotopoulos *et al*., 2006). More recently, it has been demonstrated that the blast fungus *Magnaporthe oryzae* secretes the fungal protein MoAO1, which targets the rice AOs, in order to control the apoplastic redox state and support virulence through promoting ROS accumulation (Hu *et al*., 2022). In particular, MoAO1 binds to OsAO3 and OsAO4, decreasing their enzymatic activities and resulting in a reduced apoplastic redox state that is more conducive to pathogen invasion. From the evolutionary point of view, the authors revealed that sequence polymorphism variations in MoAO1 can enable the rice AOs to reduce their binding affinities to the viral MoAO1, thus attenuating the virulence of *M. oryzae*. These findings suggest that apoplastic AO, which is in the first line of defense against pathogen infection, may be a target for evolutionary adjustments to adapt to host specialization.

Apart from its important role in mediating plant responses to pathogen attacks, AO has been reported to play a positive role in symbiotic mutualistic interactions with microorganisms. Early studies in *L. japonicus* highlighted the unexpected role of AO in mutualistic plant–microbe interactions (Balestrini *et al*., 2012). In particular, AO seems to serve as an O_2_ remover during nodule formation in roots, enabling nitrogen fixation by rhizobia, as well as being a key component of arbuscular mycorrhizal fungi colonization. Tissues colonized by fungi undergo total metabolic reprogramming, with *AO* being remarkably up-regulated, as a key component of the signaling mechanism in arbusculated cells originating from cortical cells (Volpe *et al*., 2013). A remarkable induction of *AO* expression was also evident on inoculation of tomato seedlings with a PGPR strain genetically characterized as *Pseudomonas putida* (Papadopoulou *et al*., 2023). Inoculated plants were more tolerant to drought, suggesting that induced *AO* transcript levels are putatively implicated in the priming mechanism mediated by PGPR to induce tolerance to forthcoming stress stimuli. Based on this observation, AO can be better regarded as a signaling molecule regulating O_2_ concentration rather than as an eliminator of a potentially valuable antioxidant such as AsA (De Tullio *et al*., 2007).

The role of AO in defense against insects has been overlooked so far, probably owing to the lack of supporting evidence for a potential role of apoplastic AsA oxidation in the early studies. Specifically, in experiments with caterpillars and grasshoppers feeding on poplar, no remarkable differences were found in the consumption and growth rate, or in nutritional indices, between control poplar and the *AO*-overexpressing genotypes, suggesting that *AO* cannot act as a defense component against insects, at least in poplar (Barbehenn *et al*., 2008). Nonetheless, a possible role for AsA oxidation in other plant and insect species cannot be ruled out, since AsA is an essential dietary nutrient and a prominent antioxidant for most insect herbivores, while, at the same time, it controls the efficacy of plant defense systems and alters the susceptibility of insects to their natural enemies (Goggin *et al*., 2010).

## Hormonal-mediated regulation of ascorbate oxidase and *vice versa*

Several lines of evidence demonstrate fundamental links between AO activity or *AO* expression and hormones, particularly IAA, JA, and SA (Pignocchi and Foyer, 2003; Pignocchi *et al*., 2003; 2006; Fotopoulos *et al*., 2006; Sanmartin *et al*., 2007). As mentioned above, AO is a key determinant of the apoplastic AsA redox state and, conceivably, the redox state due to the absence of GSH and NAD(PH) from the apoplast (Foyer and Noctor, 2005). Hence, the redox equilibrium controlled by AO serves as a strong signal regulating both antioxidant and hormonal metabolism (R. Stevens *et al*., 2018). However, a causal relationship remains to be established, as the regulatory signal seems to be species- and tissue-dependent. Early studies reported that IAA treatments enhanced AO activity and *AO* transcript levels in maize root quiescent center (Esaka *et al*., 1992), tobacco (Pignocchi *et al.,* 2003), and cotton (Xin *et al*., 2016), whereas AO can also increase IAA catabolism in the root tip via oxidative decarboxylation (Kerk *et al*., 2000). In turn, IAA accumulation can stimulate plant growth, but surprisingly, this increase was evident only in wild-type plants and not in *AO*-overexpressing lines (Pignocchi *et al*., 2006). Furthermore, gene expression studies in tomato *AO* RNA-silencing lines revealed that a broad number of auxin-responsive genes were induced in both leaf and fruit tissues (Garchery *et al*., 2013). Promoter analysis of the *AO* gene revealed an auxin-responsive element, with a *cis*-acting (TGA) element responsible for IAA regulation, in pumpkin (Kisu *et al*., 1997) and in cotton (Xin *et al*., 2016). Therefore, it can be assumed that this auxin-responsive element regulates the loop between *AO* transcription and IAA levels. To this end, when there is an excess of oxidized AsA in the apoplast (low AsA redox state), or when AsA oxidation is up-regulated in transgenic plants, there is a loss of IAA response and sensitivity, leading to slower rates of growth (R. Stevens *et al*., 2018). It is well known that AO exhibits highest activity in areas of cell expansion (Smirnoff, 2018). Additionally, being a cofactor of dioxygenases, AsA is presumed to be implicated in the catabolism of IAA. Consequently, any rapid modification in the apoplastic AsA redox state can indirectly influence IAA levels.

By contrast, SA, which is generally regarded as a growth suppressor, can decrease *AO* transcript levels and inhibit growth in tobacco plants (Pignocchi *et al*., 2003), whereas JA, a growth stimulator, is able to induce the accumulation of *AO* transcript levels in melon seedlings (Sanmartin *et al*., 2007). Interestingly, sugar beet sprayed with AO displayed a systemic resistance to the cyst nematode *H. schachtii*, which correlated with the elevated accumulation of both JA and SA (Singh *et al*., 2020a). Similar results were obtained in rice, highlighting that the lipoxygenase-dependent JA biosynthetic pathway can be activated following AO spraying (Singh *et al.*, 2020b). From the opposite angle, treatments with methyl jasmonate also reduced AO activity and increased DHAR activity, leading to elevated AsA levels and delayed the development of chilling injury symptoms in loquat fruit (Cai *et al*., 2011), providing further evidence that the hormone-mediated regulation of *AO* is species-dependent.

AsA is required for the activities of a broad range of enzymes, including 9-*cis*-epoxycarotenoid dioxygenase, the key gene in ABA biosynthesis (Foyer *et al*., 2020). Furthermore, in Arabidopsis *vtc2*-deficient mutants, repressed growth was linked to both ABA and ethylene (Plumb *et al*., 2018), whereas both molecules seem to act antagonistically to control AsA biosynthesis via the transcription factors ETHYLENE-INSENSITIVE (EIN3) and ABA INSENSITIVE4 (ABI4) (Yu *et al*., 2019). Although the association between AsA and ABA has been well justified, the interplay between AO and ABA remains largely untapped. In early studies with transgenic tobacco overexpressing *AO*, a substantial increase in ABA levels was reported, pointing to a shift in ABA metabolism due to *AO* overexpression, and this led to the closure of stomata (Fotopoulos *et al*., 2008). In *A. nanus*, promoter analysis identified 13 *AO* genes carrying the *cis*-acting elements involved in ABA responses, suggesting that AO may participate in ABA-mediated stress responses (Zhu *et al*., 2023). By contrast, Singh *et al*. (2020a) demonstrated that spraying sugar beet plants with AO efficiently induced systemic resistance to nematodes in the root through several phytohormones such as ethylene, JA and SA, but not through ABA, questioning the direct link between AO activity and ABA levels, which merits further investigation. Conclusively, these findings support the notion that AO has an active role in orchestrating responses to abiotic and biotic stresses not only through controlling the apoplastic redox homeostasis but also through hormonal signaling.

## Roles of ascorbate oxidase during fruit development and ripening

**Fig. 2.**
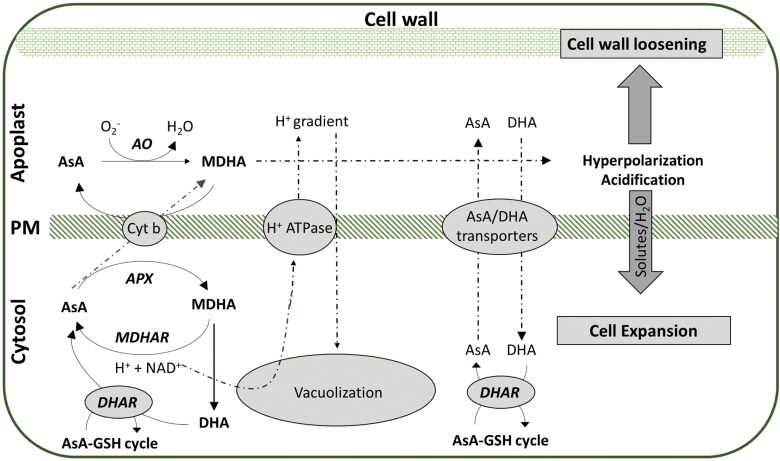
A proposed model of the action of ascorbic acid (AsA) and ascorbate oxidase (AO) enzyme activity in cell expansion and cell wall loosening. AsA is transported to the apoplast by plasma membrane-associated transporters, where it can be oxidized to monodehydroascorbate (MDHA) by AO. Cytochrome b561 (cyt b) is able to transfer electrons directly across the plasma membrane from cytoplasmic AsA to apoplastic MDHA. The regeneration of MDHA in the apoplast could lead to acidification and hyperpolarization that, in turn, accelerate vacuolization into the cytosol and cell loosening, hence stimulating cell expansion. In the cytosol, MDHA and dehydroascorbate (DHA) can be recycled back to AsA through the AsA–glutathione (GSH) cycle. Dashed lines indicate flow direction. APX, ascorbate peroxidase; DHAR, dehydroascorbate reductase; MDHAR, monodehydroascorbate reductase; PM, plasma membrane.

A growing body of evidence suggests that AsA is involved in cell division and expansion, as it is predominantly found in actively growing tissues (Liso *et al.*, 1984; Green and Fry, 2005; Smirnoff, 2018). Accordingly, several reports have demonstrated that *AO* transcription and AO activity are elevated in fast-growing tissues, including ovaries and both immature and ripe fruits (Moser and Kanellis, 1994; Diallinas *et al*., 1997; Kato and Esaka, 2000; Al-Madhoun *et al*., 2003; Sanmartin *et al*., 2007; Chatzopoulou *et al*., 2020). Nonetheless, the fate of *AO* transcription after these early stages in fruit development and ripening, as well as its function, seems to be species-dependent. Within the ripe fruit, AO is mostly active in the cell wall fraction of the outer mesocarp (Diallinas *et al*., 1997; Sanmartin *et al*., 2007). In transgenic melon with reduced *AO* transcription, AO activity was severely reduced in this area, correlating with a decrease in fruit size (Chatzopoulou *et al*., 2020). Recently, in long shelf-life (LSL) tomatoes, which have improved storage properties, AsA oxidation was stimulated through the up-regulation of *AO* orthologs compared with short shelf-life tomatoes, at the pink stage of ripening, and this also correlated with lower AsA content (Mellidou *et al*., 2023). Since *AO* transcript levels are regulated by growth promoters and suppressors (Pignocchi *et al*., 2003; Sanmartin *et al*., 2007), as previously discussed, it has been assumed that AO activity controls cell expansion (Lin and Varner, 1991; Smirnoff, 2000). A proposed model of the function of AO in cell wall expansion is provided in Fig. 2. In the apoplast, the AO-mediated oxidation of AsA produces MDHA, which, in turn, receives electrons directly from cytoplasmic AsA through cytochrome b561 (cyt b) (Davey *et al*., 2000). In this case, AsA oxidation can induce the hyperpolarization of the plasma membrane and the acidification of the apoplast, triggering solute transport to the cytoplasm (Gonzalez-Reyes *et al*., 1994). It is believed that, through this mechanism, the osmotic pressure is altered, facilitating water transport into the cytoplasm and vacuole, thereby promoting cell enlargement. Recent findings in melon underpin this theory, although further experimental evidence is required. In particular, silencing of *AO* has been shown to reduce the apoplastic MDHA and DHA contents, resulting in transgenic fruits with reduced growth and smaller fruit cell size, probably through the deactivation of H^+^-ATPases and the blockage of hyperpolarization of the plasma membrane, which eventually prevents expansion of the cytoplasm and vacuole (Chatzopoulou *et al*., 2020). Taken together, these findings reinforce the current notion that AO has a central role in regulating cell expansion and fruit growth during fruit ripening, especially in cucurbits.

Climacteric fruits such as melon and tomato are characterized by a burst in ethylene production followed by an increase in cellular respiration during ripening (Giovannoni *et al*., 2017). As AsA serves as an enzyme cofactor of 1-aminocyclopropane-1-carboxylate oxidase (ACO), which catalyzes the last step in the ethylene biosynthetic pathway, it is involved in several vital developmental processes, such as ripening Mellidou and Kanellis, 2023; Arabia *et al*., 2024). It has been hypothesized that the reduction in *AO* transcription and activity during fruit ripening, which leads to higher AsA levels, as occurs in melon (Chatzopoulou *et al*., 2020), would release AsA for ACO activity and also stimulate respiration by enhancing O_2_ availability (De Tullio *et al*., 2004). This hypothesis has been confirmed in recent studies with transgenic melon plants that have elevated AsA levels due to *AO* silencing (Chatzopoulou *et al*., 2020), suggesting that ethylene may be transcriptionally and antagonistically modulated by *AO*, although the exact ACO localization and the actual site of ethylene biosynthesis remain unclear (Houben and Van de Poel, 2019). This putative and intriguing interplay between AO and ethylene, in which AO activity decreases and consequently AsA increases, can be attributed to the enhanced supply of H^+^ to 1-aminocyclopropane-1-carboxylate (ACC) through ACO (De Tullio, 2020) and/or to the modification of the apoplastic redox signaling, as AsA could possibly act as a signaling molecule itself, irrespective of ROS (De Tullio, 2020; Foyer *et al*., 2020). Apoplastic AsA has long been regarded as a molecule implicated in cell wall loosening, promoting cell expansion and fruit softening, owing to its ability to act as either a pro-oxidant or an antioxidant (Green and Fry, 2005). This has been confirmed in our recent studies in tomato fruit, with short shelf-life varieties containing higher amounts of AsA (Mellidou *et al*., 2023). In another study, transgenic melon fruits with suppressed *AO* transcription and elevated levels of AsA were less firm than wild-type fruits (Chatzopoulou *et al*., 2020). This loss of firmness in *AO*-silenced fruits was associated with an earlier boost in ethylene production, leading to the up-regulation of several cell wall modifying genes, including an expansin (*CmEXP1*) and a polygalacturonase (*CmMPG1*). Notably, transgenic fruits also had reduced levels of DHA during ripening; this molecule is able to prevent cell wall protein cross-linking, and to remove Ca^2+^ from the cell wall by enhancing oxalate formation (Green and Fry, 2005). In melon, AO can directly contribute to fruit softening through both ethylene-dependent and -independent pathways. Similar results have been reported in tomato *AO* RNAi lines, highlighting that the transport of DHA/AsA across the plasma membrane can facilitate vacuolation and cell wall loosening (Garchery *et al*., 2013) (Fig. 2). Nevertheless, in another study employing the same *AO* RNAi lines, no distinctive differences in fruit firmness were observed compared with either wild-type or *AO*-overexpressing lines (Abdelgawad *et al*., 2019), suggesting that, at least in tomato, AO may not clearly target the cell wall, or there may be other, more complicated mechanisms orchestrating both ethylene-dependent and -independent networks of fruit cell wall integrity. Another possible explanation for this inconsistency between melon and tomato could be that in tomato, the function of AO in the cell wall may be supported by the 50% of AO activity that remains in the transgenic lines compared with wild-type plants, whereas in melon, the reduction in AO activity is so great (only 10% of the activity in the wild type at the ripe stage) that cell wall integrity cannot be maintained.

Interestingly, tomato RNAi plants with reduced AO activity were found to have higher fruit yield and sugar content upon drought (Garchery *et al*., 2013). One conceivable reason is that the low apoplastic sucrose levels in the transgenic fruits might enhance the import of sucrose from the phloem more efficiently owing to the inverse relationship between sucrose levels in fruits and the rate of sucrose import. Consequently, the sucrose is rapidly converted to fructose and glucose through the course of fruit ripening, thereby elevating the sugar content in *AO* RNAi tomato fruit. The variation in sugar content observed in *AO* RNAi tomatoes could potentially be attributed to a higher apoplastic AsA redox state compared with the wild type. Although the specific mechanisms governing carbon allocation in this context are yet to be fully understood, it is well documented that when the concentration of sucrose increases, ripening is accelerated, as occurs in strawberry (Luo *et al*., 2019) and tomato (Li *et al*., 2016). Apart from sugar content, *AO* suppression has been found to stimulate transcript levels of a melon alcohol acyltransferase, contributing to the synthesis of volatile esters that provide the typical fruit aroma (Galaz *et al*., 2013), presumably due to the induction of ethylene biosynthesis (Chatzopoulou *et al*., 2020).

Putting these observations aside, the fate of fruit AsA upon alterations in *AO* transcription and protein content during ripening is not always clear and seems to be dynamically regulated in a species- and stage-dependent manner. In particular, as expected, in transgenic melon fruits with reduced AO activity, apoplastic AsA levels were significantly enhanced at pre-climacteric stages compared with wild-type fruits, accompanied by lower DHA content and the up-regulation of AsA biosynthetic and recycling genes (Chatzopoulou *et al*., 2020). These novel findings indicated that at least in melon fruit, lowering AO activity can remarkably modulate the apoplastic AsA redox state, suggesting the potential for enhancing the size of the AsA pool through this unexpected route (Mellidou *et al*., 2021). In tomato fruits, *AO* transcript levels are high at the early stages prior to the climacteric stage, and they then drop as ripening proceeds (Ioannidi *et al*., 2009). In contrast, mutation of the other enzyme responsible for the oxidation of the AsA pool (but in the symplast), ascorbate peroxidase, resulted in a reduction of AsA content during the later stages of fruit ripening in tomato (Do *et al*., 2022), suggesting that AsA oxidation has a different role to perform based on the pathway in which it takes place.

From a different perspective, tomato fruits at the red ripe stage from genotypes with elevated AsA content showed a reduction in *AO* expression levels, as well as an induction of AsA-recycling transcript levels, compared with fruits with a low-AsA genotype (Mellidou *et al*., 2023). In view of the above, it is clear that AO exhibits cultivar and stage-dependent regulation. Surprisingly, however, transgenic tomato fruits with increased AsA contents resulting from overexpression of the key AsA biosynthetic gene (*SlGGP1*) also exhibited higher transcript levels of several *AO* orthologs at the pink stage of ripening (Koukounaras *et al*., 2022). This observation clearly points to a feedback regulatory mechanism of the AsA pool size that could be essential for the constant fine-tuning of the redox state at stages before the red ripe stage. Quantitative trait locus (QTL) analysis in apple (Mellidou *et al*., 2012) and strawberry (Muñoz *et al*., 2023) identified major QTLs for fruit AsA contents co-locating with *AO* orthologs. However, in the case of apple, these QTLs were not stable over the years, whereas in the case of strawberry, the candidate *AO* gene was not differentially expressed in lines with contrasting AsA levels, revealing that *AO* cannot be the key determinant of AsA pool size. In a recent study by Bulley *et al*. (2021), it was demonstrated that enhancement of the AsA pool in Arabidopsis via feeding plants with AsA precursors did not lead to a notable alteration in *AO* expression levels but instead stimulated *MDHAR* and *DHAR* transcription. Given these findings, it is evident that the AsA redox state is constantly under tight control, and any changes in the transcript levels of these genes are short-lived to reach the optimal redox balance. Conversely, in persimmon fruits, there was a strong positive correlation between five *AO* genes and AsA content in different genotypes (*P*<0.01) throughout the course of ripening, further supporting the notion that the AO regulatory mode may be dependent on both the species and the stage of ripening. Interestingly, in recent studies of tomato *AO* RNAi lines, their fruit transcriptome was inversed compared with those of the equivalent *MDHAR* and l-galactono-1,4-lactone dehydrogenase (l-*GLDH*) lines; furthermore, it did not correlate with the fruit proteome and metabolome of these lines (R.G. Stevens *et al*., 2018). As a result, it can be assumed that AO, along with the AsA redox state, may be implicated in the transcriptional response of AsA and downstream elements, to attenuate the severity of oxidative damage.

## Conclusion

Although the enzyme AO was discovered early and has been well characterized in several plant species, there are still many fundamental aspects of its regulatory role in plant development, fruit growth, and ripening, as well as in plant responses to biotic and abiotic stimuli, that need to be better investigated. Overall, the expression of *AO* is tightly regulated, influenced by growth promoters and suppressors (hormones), as well as by developmental and environmental stimuli, and also seems to be species dependent. Any alterations in *AO* expression can affect AsA levels, potentially influencing plant tolerance to stress. However, contrary to the initial consensus that *AO* expression merely weakens plant defense, an accumulating body of evidence suggests that its contribution as an orchestrator of plant responses is more complex, while its link with O_2_ sensing could possibly serve as a delicate target to make plants more capable of sensing environmental stimuli. On the other hand, the correlation between AO activity and plant growth rate or fruit ripening is still intriguing, at least in species other than cucurbits. The high AO activity in the species of the Cucurbitaceae family may corroborate its selection during the domestication process to support the rapid fruit growth rate and the large fruit of cucurbits, which is not always the case in other fruit species, such as tomato. Transcription factors and small RNAs regulate *AO* expression, while discrepancies between AO enzyme activity and *AO* transcript levels suggest the existence of post-translational or isoform-specific regulation. Epigenetic marks, such as DNA methylation and histone modifications, also have an impact on *AO* expression under stress conditions and during development/ripening, showcasing the intricate regulatory networks governing the oxidation of the AsA pool. Hence, understanding the multifaceted regulatory mechanisms of *AO* in different plant species, tissues, and ripening stages, including epigenetic and post-transcriptional processes, will be fundamental to fill the current gaps concerning its function, going beyond the common notion of the battle between ‘beneficial’ antioxidants and ‘harmful’ ROS.
